# Cancer survivors who fully participate in the PROFILES registry have better health-related quality of life than those who drop out

**DOI:** 10.1007/s11764-019-00793-7

**Published:** 2019-09-06

**Authors:** Imogen Ramsey, Belle H. de Rooij, Floortje Mols, Nadia Corsini, Nicole J. E. Horevoorts, Marion Eckert, Lonneke V. van de Poll-Franse

**Affiliations:** 1 Rosemary Bryant AO Research Centre, School of Nursing and Midwifery and UniSA Cancer Research Institute, University of South Australia, Adelaide, Australia; 2 The Netherlands Comprehensive Cancer Organization, Utrecht, the Netherlands; 3 Department of Medical and Clinical Psychology, CoRPS—Center of Research on Psychology in Somatic Diseases, Tilburg University, Tilburg, the Netherlands; 4 Division of Psychosocial Research and Epidemiology, the Netherlands Cancer Institute, Amsterdam, the Netherlands

**Keywords:** Cancer, Dropout, Patient-reported outcomes, Quality of life, Survivorship, Bias, Attrition

## Abstract

**Purpose:**

Attrition and subsequent missing data pose a challenge in longitudinal research in oncology. This study examined factors associated with attrition in the PROFILES registry, and its impact on observed health-related quality of life (HRQOL) estimates.

**Methods:**

Sociodemographic, clinical, and HRQOL data were collected annually from a cohort of 2625 colorectal cancer survivors between 2010 and 2015. Participant characteristics according to time of dropout were compared using analysis of variance and chi-square tests. Predictors of attrition were examined in logistic regression analysis. Multilevel linear mixed models were constructed to investigate associations between attrition and HRQOL over time.

**Results:**

Participants who dropped out were more likely to be female (OR = 1.23, CI = 1.02–1.47), older (OR = 1.20, CI = 1.09–1.33), less educated (OR = 1.64, CI = 1.30–2.11), and to have depressive symptoms (OR = 1.84, CI = 1.39–2.44) than full responders, and less likely to have high socioeconomic status (OR = 0.74, CI = 0.61–0.94). Participants who dropped out earlier reported significantly worse HRQOL, functioning, and psychosocial symptoms, which declined at a steeper rate over time, than full responders.

**Conclusions:**

Cancer survivors’ HRQOL may be overestimated in longitudinal research due to attrition of the most unwell participants.

**Implications for Cancer Survivors:**

Cancer survivors with the poorest health are at risk of dropping out of PROFILES and possibly withdrawing from other activities. Optimizing participation in PROFILES—a potential mechanism for providing information and access to support—is an avenue for keeping this group engaged.

**Electronic supplementary material:**

The online version of this article (10.1007/s11764-019-00793-7) contains supplementary material, which is available to authorized users.

## Background

Patient-reported outcomes (PRO) are patients’ self-reports about the impacts of a health condition on functioning, symptoms, and health-related quality of life (HRQOL) as well as experiences of treatment and care. PRO data can support the provision of patient-centred care by informing decision-making at the individual level, driving quality improvement at a system level, and determining factors that influence patient outcomes on a population level [1]. Missing data poses a challenge for PRO research, particularly as it relates to attrition (i.e. when a participant drops out and is never observed again) [2]. Despite its implications for data quality, analysis, and interpretation, the mechanisms of attrition in population-based PRO research are not well understood [2].

The Netherlands PROFILES registry is a unique system for comprehensive population-level PRO monitoring, which seeks to understand the burden and trajectory of outcomes experienced by cancer survivors post-treatment [3]. Since 2008, PROFILES has collected longitudinal PRO data from over 20,000 cancer survivors, with participation rates similar to or higher than comparable observational studies [4–6]. Through population-level reach, PROFILES provides a novel way to surmount some of the challenges associated with recruiting and retaining post-treatment cancer survivors in longitudinal clinical research, allowing greater external validity and generalizability [7]. Other advantages include data linkage with the Netherlands Cancer Registry (NCR), which records clinical and sociodemographic information about all individuals newly diagnosed with cancer in the Netherlands. However, this type of observational research requires dedicated participants who are able and willing to participate long term.

Attrition is a ubiquitous problem in longitudinal research [8]. Because it may be selective (e.g. due to declining health or death), attrition can bias the representativeness of the sample [8]. Factors associated with attrition in oncology trials include death, symptom burden, illness, advanced disease, increased age, low socioeconomic status, and being from a minority group [9–11]. A cross-sectional study comparing invited cancer survivors who did not participate in PROFILES with those who did found that non-participants had lower survival and lower estimated HRQOL than participants [12], but it is not known whether similar differences exist between those who participate long term and those who drop out. Furthermore, there have been few attempts to document the pattern of and reasons for attrition in population-based cohort studies of cancer survivors generally [2, 13]. Understanding how, why, and who is likely to participate in this context has important implications for interpreting findings from PROFILES, estimating sample sizes, and improving participant retention in future studies.

This study aimed to determine the rate of and factors associated with attrition among colorectal cancer survivors participating in the largest cohort of the PROFILES registry, and to assess the impact of attrition on observed HRQOL outcomes over time. This population provides a useful case to examine  the challenges of conducting longitudinal PRO research; colorectal cancer is the third most common cancer in Europe and the world, and the 5-year survival rate in the Netherlands is 65% [14]. The study objectives were to (1) examine sociodemographic and clinical factors that influence the likelihood of attrition in PROFILES, and (2) investigate differences in longitudinal HRQOL, anxiety, and depressive symptoms according to time of dropout.

## Methods

### Design and setting

**Fig. 1:**
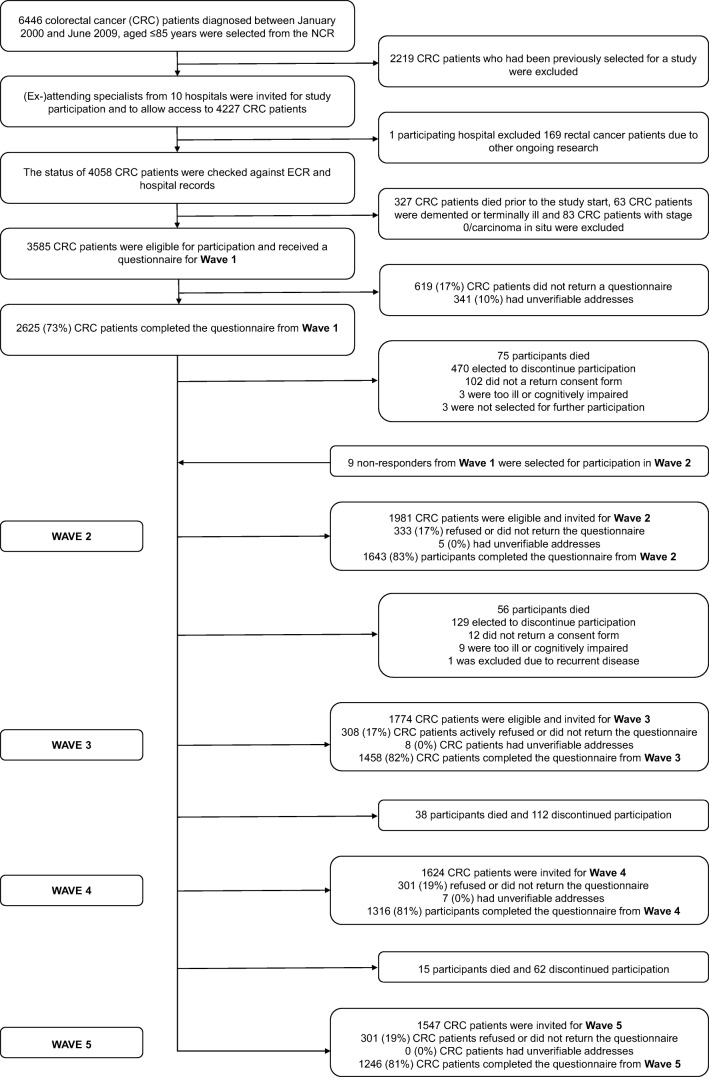
Flow chart of study participation

This study used data from PROFILES, which collects PRO from cancer survivors within a sampling frame of the NCR. The first wave commenced in December 2010 (T1) and participants received follow-up questionnaires in 2011 (T2), 2012, (T3), 2013 (T4), and 2014 (T5). Figure 1 presents an overview of study participation.

### Data collection

The data collection process has been described in detail elsewhere [3, 15]. In brief, eligible participants were informed about PROFILES via a letter from their (ex-)attending specialist, accompanied by a consent form and questionnaire or a secured link to an online form and questionnaire, with an option to request a paper version. If no response was received after 2 months, a reminder letter and paper questionnaire was sent. Participants who did not complete a questionnaire were classified as non-responders for that wave but could still be invited to subsequent waves.

### Study population

Eligible participants were individuals diagnosed with colon or rectal cancer between January 2000 and June 2009 as registered in the NCR. Individuals unable to complete a questionnaire according to their (ex-)attending specialist (i.e. cognitively impaired, too ill, or non-native speaker) or who had unverifiable addresses were excluded. Address checks were performed at each wave to verify whether the registered address corresponded with national zip code registration.

### Study measures

PRO were assessed at T1, T2, T3, and T5. The T4 questionnaire contained measures unrelated to the study outcomes reported here and was therefore considered in terms of response only. Dates of invitation and response were recorded at each wave.

### Sociodemographic characteristics

Sociodemographic data included sex, birth date, and socioeconomic status (SES). SES was determined based on residential postcode and aggregated fiscal data [16]. Marital status and education were assessed in the questionnaire.

### Clinical characteristics

Clinical data included date of diagnosis, tumour type, stage, and primary treatments. Tumour type and stage were classified according to the third International Classification of Diseases for Oncology (ICDO-3) [17] and TNM, respectively [18]. Comorbidity was assessed with the adapted Self-Administered Comorbidity Questionnaire [19]. Mortality data were obtained from the Dutch municipal personal records database and were last verified on 31 January 2018.

### Patient-reported outcome measures

The EORTC QLQ-C30 (version 3.0) was used to assess HRQOL [20]. The questionnaire contains scales for physical, role, social, emotional, and cognitive functioning; a global HRQOL scale; and symptom scales for fatigue, pain, and nausea/vomiting [20]. Items are rated on a four-point scale ranging from 1 to 4. All scores were linearly transformed to a scale of 1 to 100 as per the EORTC guidelines [21]. A summary score was calculated from the mean of 13 of the 15 QLQ-C30 scales [22].

Depressive and anxiety symptoms were assessed with the Hospital Anxiety and Depression Scale (HADS), which contains two 7-item subscales for each construct [23]. Items are scored on a four-point scale ranging from 0 to 3, with higher scores indicated higher symptom prevalence. Sum scores ranging from 0 to 21 were calculated for both subscales, and a recommended cut-off score of 8 was used to define the presence of anxiety disorders and depressive symptoms [24].

### Statistical analysis

Participants were stratified into groups based on their last completed questionnaire: dropped out after T1, dropped out after T2, dropped out after T3/T4 (combined due to aforementioned difference in the T4 assessment), and full responders (participants who completed the final assessment, regardless of intermittently missing assessments).

Sociodemographic and clinical group characteristics were compared using analysis of variance (ANOVA) and chi-square tests where appropriate. Post hoc comparisons were made using Tukey’s method. To investigate predictors of attrition, multivariable logistic regression analysis was performed with full response vs. dropout as outcomes, excluding participants with attrition due to death. Factors included as potential predictors were sex, age, partner, SES, education, comorbidity, treatment, QLQ-C30 functioning subscales, depressive symptoms, and anxiety.

Multilevel linear mixed models were constructed to investigate associations between study participation and PRO, allowing adjustment for interdependency of repeated observations within patients and correction for missing data at random [25]. This technique uses data efficiently by including incomplete cases in analysis, limiting bias, and preserving statistical power [26]. Time was analysed as a categorical predictor with four levels (T1, T2, T3, T5). Sociodemographic and clinical variables were analysed as time-invariant predictors using T1 characteristics. Overall effects were assessed comparing outcomes for full responders with participants who dropped out stratified by time of dropout. The final models were adjusted for time, age, sex, SES, education, marital status, comorbidity, disease stage, and treatment. Graphs present unadjusted means by group over time, with *p* values indicating differences between group means and slopes after adjustment. To highlight these differences, graphs are presented on a scale from 60 to 90. Sensitivity analyses excluding participants who died were performed.

Statistical tests were two-sided and considered significant if *p* < 0.05. Analyses were performed in SAS version 9.4. Clinically relevant differences were determined using evidence-based guidelines for interpretation of the QLQ-C30 between groups, which provide estimates for trivial, small, medium, and large mean differences [27].

## Results

### Attrition rate

The survey was completed by 73% of invited participants at T1 (*n* = 2625). Of those that were eligible and invited at each wave, the proportion of participants who completed that wave was 83% at T2 (*n* = 1643), 82% at T3 (*n* = 1458), 81% at T4 (*n* = 1316), and 81% at T5 (*n* = 1216). Table 1 shows the number of participants who dropped out after each assessment. Total attrition was 53% (*n* = 1388). In most cases the reason was unknown (*n* = 1174, 83%). Death accounted for 13% of attrition overall (*n* = 184), 1% of participants had unverifiable addresses during follow-up (*n* = 20), and < 1% were unable to continue participation due to illness or cognitive impairment (*n* = 12).

**Table 1:** Participant characteristics by time of dropout

Characteristics		Non-participants^a^ *N* = 951	Dropout T1 *N* = 856	Dropout T2 *N* = 219	Dropout T3/T4 *N* = 313	Full responders *N* = 1246	*p* value
Sex							< 0.001
	Male	458 (48%)	432 (50%)	119 (54%)	177 (57%)	723 (58%)	
	Female	493 (52%)	424 (50%)	100 (46%)	136 (43%)	523 (42%)	
Age (years), *M* (SD)		70.9 (11.2)	71.4 (9.3)	70.5 (9.6)	69.3 (9.8)	67.9 (9.3)	< 0.001
Age (years)							< 0.001
	< 59	144 (15%)	105 (12%)	33 (15%)	48 (15%)	225 (18%)	
	60–69	230 (24%)	209 (24%)	60 (27%)	108 (35%)	473 (38%)	
	70–79	355 (37%)	383 (45%)	88 (40%)	112 (36%)	440 (35%)	
	80<	222 (23%)	159 (19%)	38 (17%)	45 (14%)	108 (9%)	
Partner							< 0.001
	Yes	–	600 (71%)	166 (78%)	239 (77%)	979 (79%)	
SES							< 0.001
	Low	275 (29%)	196 (23%)	45 (21%)	68 (22%)	217 (17%)	
	Medium	385 (40%)	360 (42%)	95 (43%)	136 (44%)	471 (38%)	
	High	245 (26%)	275 (32%)	71 (32%)	94 (30%)	506 (41%)	
	Unknown/care institution	46 (5%)	25 (3%)	8 (4%)	15 (5%)	52 (4%)	
Education							< 0.001
	Low	–	230 (27%)	55 (25%)	63 (20%)	172 (14%)	
	Medium	–	489 (57%)	124 (57%)	187 (60%)	768 (62%)	
	High	–	117 (14%)	31 (14%)	62 (20%)	298 (24%)	
	Unknown	–	20 (2%)	9 (4%)	1 (0%)	8 (1%)	
Clinical							Clinical
Years since diagnosis, *M* (SD)		5.3 (2.9)	5.3 (2.8)	5.0 (2.8)	5.1 (2.8)	5.1 (2.8)	0.52
Stage							0.001
	I	241 (25%)	249 (29%)	58 (26%)	87 (28%)	392 (31%)	
	II	388 (41%)	326 (38%)	78 (36%)	115 (37%)	432 (35%)	
	III	244 (26%)	209 (24%)	65 (30%)	89 (28%)	359 (29%)	
	IV	50 (5%)	56 (7%)	14 (6%)	12 (4%)	31 (2%)	
	Unknown	28 (3%)	16 (2%)	4 (2%)	10 (3%)	32 (3%)	
Chemotherapy							0.76
	Yes	251 (26%)	241 (28%)	68 (31%)	90 (29%)	373 (30%)	
Radiotherapy							0.02
	Yes	231 (24%)	232 (27%)	62 (28%)	108 (35%)	406 (33%)	
Surgery							< 0.01
	Yes	929 (98%)	839 (98%)	217 (99%)	312 (100%)	1240 (100%)	
Comorbidities							< 0.01
	0	–	183 (21%)	45 (21%)	83 (27%)	303 (24%)	
	1	–	201 (23%)	52 (24%)	71 (23%)	385 (31%)	
	2	–	170 (20%)	48 (22%)	71 (23%)	266 (21%)	
	3 or more	–	202 (24%)	60 (27%)	71 (23%)	241 (19%)	
	Unknown	–	100 (12%)	14 (6%)	17 (5%)	51 (4%)	
Died before next invitation		72 (28%)	75 (13%)	56 (31%)	53 (20%)	–	< 0.001

Note: *p* values report overall ANOVA for normally distributed continuous variables, chi-square tests for categorical variables, and Wilcoxon tests for non-normally distributed continuous variables. Means (*M*) with standard deviations (SD) were used to describe normally distributed continuous variables and frequencies with percentages were used to describe categorical variables

^a^Invited cancer survivors who declined to participate or had unverifiable addresses

### Sociodemographic and clinical characteristics

It has been previously reported that participants at T1 had a longer time since diagnosis and were significantly younger and more often male, diagnosed with stage I disease, and treated with radiotherapy than non-participants at T1 [28]. Full responders were more often male, less than 70 years old, partnered, university educated, diagnosed at stage I, and treated with radiotherapy than participants who dropped out or non-participants (Table 1). They were also more likely to have high SES and one comorbid condition, but less likely to have three or more comorbid conditions, than participants who dropped out or non-participants (Table 1). No group differences were found for years since diagnosis or receiving chemotherapy.

### Predictors of attrition

Predictors of attrition in multivariable logistic regression were being female (OR = 1.23, CI = 1.02–1.47), older age (OR = 1.20, CI = 1.09–1.33), low education (OR = 1.64, CI = 1.30–2.11), presence of depressive symptoms (OR = 1.84, CI = 1.39–2.44), and having missing education or comorbidity data (OR = 5.51, CI = 1.20–25.16; OR = 2.21, CI = 1.42–3.44) (Table 2). High SES was inversely associated with attrition (OR = 0.77, CI = 0.62–0.94) (Table 2).

**Table 2:** Factors associated with dropout during follow-up, multivariable logistic regression

		Full responders (*N* = 1246)	Dropouts^a^ (*N* = 1204)	Odds of dropout vs. full response	95% CI
Sex					
	Male	723 (58%)	611 (51%)	1.00 (ref)	
	Female	523 (42%)	593 (49%)	1.23*	1.02–1.47
Age, *M* (SD)		67.90 (9.31)	70.61 (9.63)	1.20**	1.09–1.33
Partner					
	Yes	979 (80%)	863 (73%)	1.08	0.87–1.34
SES					
	Low	217 (17%)	270 (22%)	0.99	0.76–1.23
	Medium	471 (38%)	514 (43%)	1.00 (ref)	
	High	506 (41%)	381 (32%)	0.74**	0.61–0.94
	Unknown/care institution	52 (4%)	41 (3%)	0.74	0.46–1.19
Education					
	Low	172 (14%)	304 (25%)	1.64**	1.30–2.11
	Medium	768 (62%)	692 (57%)	1.00 (ref)	
	High	298 (24%)	181 (15%)	0.87	0.69–1.09
	Unknown	8 (1%)	27 (2%)	5.51*	1.20–25.16
Stage					
	I	392 (32%)	356 (30%)	0.86	0.69–1.07
	II	432 (36%)	469 (39%)	1.00 (ref)	
	III	359 (30%)	310 (26%)	0.82	0.63–1.06
	IV	31 (3%)	40 (3%)	1.18	0.66–2.09
	Unknown	32 (3%)	29 (2%)	0.98	0.56–1.70
Treatment					
	Chemotherapy	373 (30%)	333 (28%)	1.06	0.83–1.36
	Radiotherapy	406 (33%)	336 (28%)	0.89	0.74–1.08
	Surgery	1240 (100%)	1194 (99%)	0.76	0.25–2.32
Comorbidities					
	0	303 (24%)	273 (23%)	1.29	0.98–1.67
	1	385 (31%)	288 (24%)	0.90	0.71–1.16
	2	266 (21%)	251 (21%)	1.00 (ref)	
	3 or more	241 (19%)	276 (23%)	0.96	0.73–1.25
	Unknown	51 (4%)	116 (10%)	2.21**	1.42–3.44
Depressive symptoms					
	Yes	160 (13%)	267 (22%)	1.84**	1.39–2.44
Anxiety					
	Yes	217 (18%)	255 (21%)	0.93	0.71–1.24
Physical functioning, *M* (SD)	83.7 (18.1)	77.9 (21.4)	0.94	0.87–1.00	
Role functioning, *M* (SD)	83.6 (24.6)	78.2 (28.1)	0.96	0.92–1.01	
Emotional functioning, *M* (SD)	87.5 (17.9)	84.9 (20.4)	0.98	0.92–1.05	
Social functioning, *M* (SD)	87.9 (20.5)	86.2 (23.1)	1.05	0.99–1.11	
Cognitive functioning, *M* (SD)	86.2 (19.8)	84.1 (20.7)	1.01	0.96–1.07	

Note: 285 observations were deleted due to missing values. Odds ratios for age and all EORTC functioning scales are expressed per 10-unit increase. Means (*M*) with standard deviations (SD) were used to describe normally distributed continuous variables and frequencies with percentages were used to describe categorical variables. Significant odds ratios are in italics

*p < 0.05; **p < 0.01

^a^Dropouts at any wave, excluding participants who dropped out due to death

### Study participation and HRQOL

ANOVA tests showed significant differences between the dropout groups on all functioning and symptom scales at T1 (all *p* values < 0.05; see Table S1). Pairwise comparisons indicated that participants who dropped out after T1 or T2 had statistically significantly lower baseline summary score, physical functioning, and role functioning and higher fatigue than participants who completed three or more assessments, and these differences were clinically relevant (Table S1). Full responders had significantly higher global HRQOL and emotional functioning, and less nausea than participants who dropped out after T1. They also had significantly lower anxiety symptoms than participants who dropped out after T2, and depressive symptoms than all dropout groups (Table S1).

**Fig. 2:**
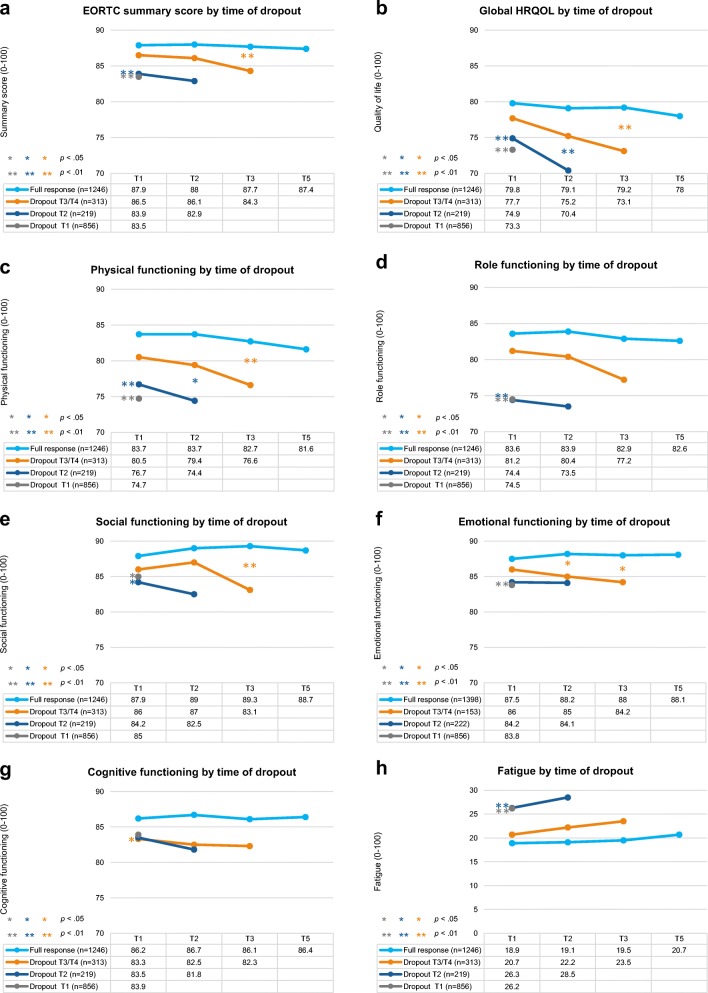

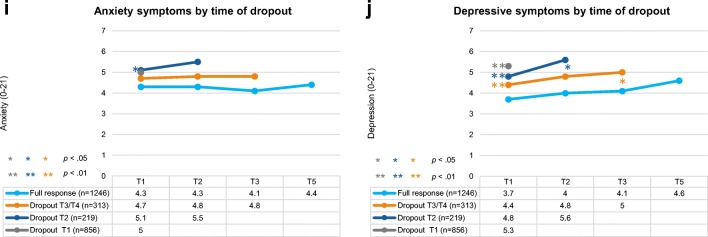
Unadjusted mean functioning scores on the EORTC QLQ-C30 (**a**–**h**) and anxiety and depressive symptoms on the HADS (**i**, **j**) according to time of dropout (range: 0–100 and 0–21, respectively). Note: EORTC QLQ-C30 scales range from 0 to 100; higher scores reflect better perceived HRQOL. HADS scales range from 0 to 21; higher scores reflect higher prevalence of anxiety and depressive symptoms. *p* values indicate significant group differences between slopes and baseline scores compared with full responders in multilevel mixed models adjusted for time, age, sex, socioeconomic status, education, marital status, comorbidity, disease stage, and treatment received

In multilevel linear mixed models adjusted for time, age, sex, SES, education, marital status, comorbidity, disease stage, and treatment, at baseline full responders had significantly higher global HRQOL, physical functioning, role functioning, social functioning, and summary score (Fig. 2a–e), and less fatigue (Fig. 2h) than participants who dropped out after T1 or T2. They also reported significantly higher emotional functioning and cognitive functioning and less anxiety symptoms than participants who dropped out after T1, and less depressive symptoms than participants who dropped out at T1, T2, or T3/T4 (Fig. 2f, g; Fig. 2i, j). There were no significant group differences for nausea or pain (not shown). The difference between the adjusted means of full responders and participants who dropped out after T1 was of small clinical importance for global HRQOL, physical functioning, role functioning, and fatigue [27].

Full responders had a more stable PRO trajectory than participants who dropped out. Over time, participants who dropped out after T2 or T3/T4 showed a steeper decline in global HRQOL and physical functioning and a steeper increase in depression than full responders (all *p* values < 0.05; see Fig. 2b, c; Fig. 2j). Compared with full responders, participants who dropped out at T3/T4 also showed steeper declines in social and emotional functioning and the summary score (all *p* values < 0.05; see Fig. 2a; Fig. 2e, f).

Sensitivity analyses excluding patients with attrition due to death showed no significant differences in PRO trajectory between full responders and participants who dropped out at T2 or T3/T4. However, there remained significant baseline differences between full responders and participants who dropped out after T1 on all PRO except for anxiety and social functioning.

## Discussion

Compared with full responders, colorectal cancer survivors who dropped out of PROFILES were more likely to be women, older, less educated, and to have depressive symptoms, and less likely to have high SES. Full responders reported better HRQOL at each assessment than participants who dropped out, with poorer outcomes generally indicating earlier dropout. Participants who dropped out after the second, third, or fourth wave showed a steeper decline in global HRQOL and physical functioning and a steeper increase in depressive symptoms  than full responders. Participants who dropped out after the third or fourth wave also showed a steeper decline in social functioning, emotional functioning, and the summary score. Sensitivity analyses suggested this trend was driven by mortality, although first wave dropouts still reported worse initial HRQOL than full responders, unrelated to mortality.

Non-response and attrition have consistently been associated with sociodemographic factors including low education and SES in longitudinal studies with cancer patients [4, 11] and general populations [29–31] and the link between low SES and poor health—another predictor of attrition—is well established [30–33]. Cancer patients with low SES are often underrepresented in clinical trials [4, 34, 35], in some instances even after accounting for education and comorbidity [35]. The higher rate of dropout among women in our sample was not unexpected, given that men are more likely to participate in PROFILES than women [12]. This is contrary to evidence that participation in population-based research is generally higher among women than men [36, 37] although studies have also observed the opposite [38, 39]. The results also differ from numerous studies with cancer populations that did not find associations between sex and participation [4–6] or attrition [9–11]. An explanation may be provided by growing evidence for sex-based differences in response to anticancer treatments, showing that women experience higher incidence of toxicity and adverse reactions than men [40, 41]. It is possible that women in our sample experienced more treatment-related symptoms than men, which made them more likely to discontinue participation. Further research is needed to give insight into sex differences in cancer and the resulting impact on research participation and other behaviours.

Associations between older age and attrition have been reported in clinical cancer trials [34], and linked with cognitive impairment and poorer health [42]. Contrary to evidence demonstrating a link between higher prevalence of health-related problems and attrition, having a higher number of comorbid conditions was not associated with dropout [31, 38]. Cancer stage did not predict likelihood of full response, although other studies have found that patients with more advanced cancer are more likely to drop out [11].

Participants who dropped out reported higher prevalence of depressive symptoms, which increased more steeply over time, compared with full responders. Epidemiologic studies have found underestimation of psychiatric disorders due to non-participation [31, 43] although the relationship between anxiety, depression, and research participation among cancer survivors is understudied. Depression frequently appears to be associated with reduced physical functioning and cancer-related symptoms including fatigue and pain [44, 45]; all of which may limit a person’s willingness or capacity to participate in research.

Our results demonstrate that participants who drop out of PROFILES have significantly lower (statistically and clinically) HRQOL than those who participate until the final assessment. Independent of mortality, first wave dropouts reported worse HRQOL at baseline than full responders, which could be explained by cancer or other illness hindering study participation. Given the recent finding that PROFILES participants survive longer than non-participants [12], our results reinforce that cancer survivors with the poorest health are likely to be underrepresented in population-based research. The problem of selection bias in PRO research has been highlighted in studies of ovarian and head and neck cancer patients undergoing or up to 1 year post-treatment [2, 13, 46], where participants with the lowest baseline HRQOL were more likely to drop out. After separating participants with attrition due to death, one study observed fewer group differences at baseline after adjustment for sociodemographic and clinical factors [47]. With 5-year follow-up and a large population-based sample allowing subgroup analysis, our study builds upon this work, providing longer-term insight into the impact of attrition on HRQOL estimates. Since dropout may be an indicator of poorer health as well as withdrawal from other activities, access to PROFILES participation data could help clinicians identify patients at risk and refer them to clinical interventions.

It is likely that participation was influenced by factors beyond those measured. Participation in population-based research is in decline, possibly owing to increased requests to participate in research, heightened demands and complexity of research procedures, and a general decrease in volunteerism [29]. Characteristics of longitudinal studies with high retention include individually tailored retention strategies, iterative adaptation and refinement of retention processes, and innovative and persistent research teams [48]. Our results suggest that efforts to address representativeness and retention in PROFILES might best be directed towards recruitment and the first follow-up. To increase participation, PROFILES has recently implemented phone calls to consenting patients to explain the study. This strategy appears to have improved the initial response rate, but it is unknown whether it will help to minimize attrition. Formal evaluation of this approach is recommended.

To our knowledge, no studies have explored the impact of attrition on HRQOL in cancer survivors using a longitudinal and population-based cohort of this size. Study merits include the sampling frame and availability of objective sociodemographic and clinical data on participants and non-participants. Limitations include the lack of data on reasons for non-response, which could facilitate a better understanding of attrition in this population. Data on participant’s racial and ethnic backgrounds were also lacking. Given that individuals from racial minorities are underrepresented in research [49] and less likely to participate in palliative oncology trials [9], this information should be considered. Because clinical data from the NCR was only available at diagnosis, a lack of information about recurrence and disease progression was another limitation. Finally, because all participants were colorectal cancer survivors, the results cannot be generalized beyond this group. Due to differences in the timing and methods of data collection between different PROFILES cohorts, we elected to focus this investigation on one sample. Examining whether patterns of attrition vary between cohorts of cancer survivors participating in PROFILES is an avenue for future investigation.

The study findings suggest that further investigation of statistical methods to adjust for non-response and attrition in longitudinal HRQOL studies is warranted. Transparent reporting of participation and justification for how missing data were handled in future studies will facilitate interpretation. Although attrition is not fully preventable, strategies designed to retain participants [48, 50] particularly those at higher risk of attrition could improve representativeness. Financial incentives have been repeatedly associated with higher participant retention in population-based cohort studies [51] and retention rates have been found to increase with the value of monetary incentive offered [50, 51]. Tailored strategies including regular newsletters and personalized reminders, non-financial incentives, educational discussion forums, and annual events have been successfully implemented in longitudinal cohort studies with high retention rates [48]. It is hypothesised that these approaches may promote participant engagement, reinforce study benefits and identity, strengthen staff-participant relationships, and foster a sense of community, although rigorous evaluation of their (cost-)effectiveness is lacking [48]. Evidence suggests that retention rates increase with the number of strategies used [50] and therefore using a combination of methods appropriate to the study population and context is recommended.

Our findings support and expand upon other longitudinal studies in oncology showing a selection bias, whereby loss of participants due to death or illness during follow-up produces overestimates of HRQOL [13, 46, 47]. Cancer survivors with the poorest health are at the highest risk of dropping out of PROFILES and therefore of withdrawing from other activities too, including those that may benefit well-being. Thus, optimizing participation in PROFILES—a potential mechanism for providing information and access to support—is an avenue for keeping this at-risk group engaged. Understanding the biasing effects of selective attrition on PRO will help to contextualize findings from PROFILES and inform strategies for recruitment, retention, and analysis in population-based research.

## Electronic supplementary material

ESM 1 (DOCX 15 kb)
