# Correction to: Cancer survivors who fully participate in the PROFILES registry have better health-related quality of life than those who drop out

**DOI:** 10.1007/s11764-019-00813-6

**Published:** 2019-11-21

**Authors:** Imogen Ramsey, Belle H. de Rooij, Floortje Mols, Nadia Corsini, Nicole J. E. Horevoorts, Marion Eckert, Lonneke V. van de Poll-Franse

**Affiliations:** 1 Rosemary Bryant AO Research Centre, School of Nursing and Midwifery and UniSA Cancer Research Institute, University of South Australia, Adelaide, Australia; 2 The Netherlands Comprehensive Cancer Organization, Utrecht, the Netherlands; 3 Department of Medical and Clinical Psychology, CoRPS—Center of Research on Psychology in Somatic Diseases, Tilburg University, Tilburg, the Netherlands; 4 Division of Psychosocial Research and Epidemiology, the Netherlands Cancer Institute, Amsterdam, the Netherlands

**Correction to: Journal of Cancer Survivorship**

10.1007/s11764-019-00793-7

The original version of the article contains a mistake in figure 2 labeling.

The part labels of figure 2i and 2j is incorrectly labelled as 2a and 2b. The correct figure 2 is given below.

The original article has been corrected.

**Fig. 1:**
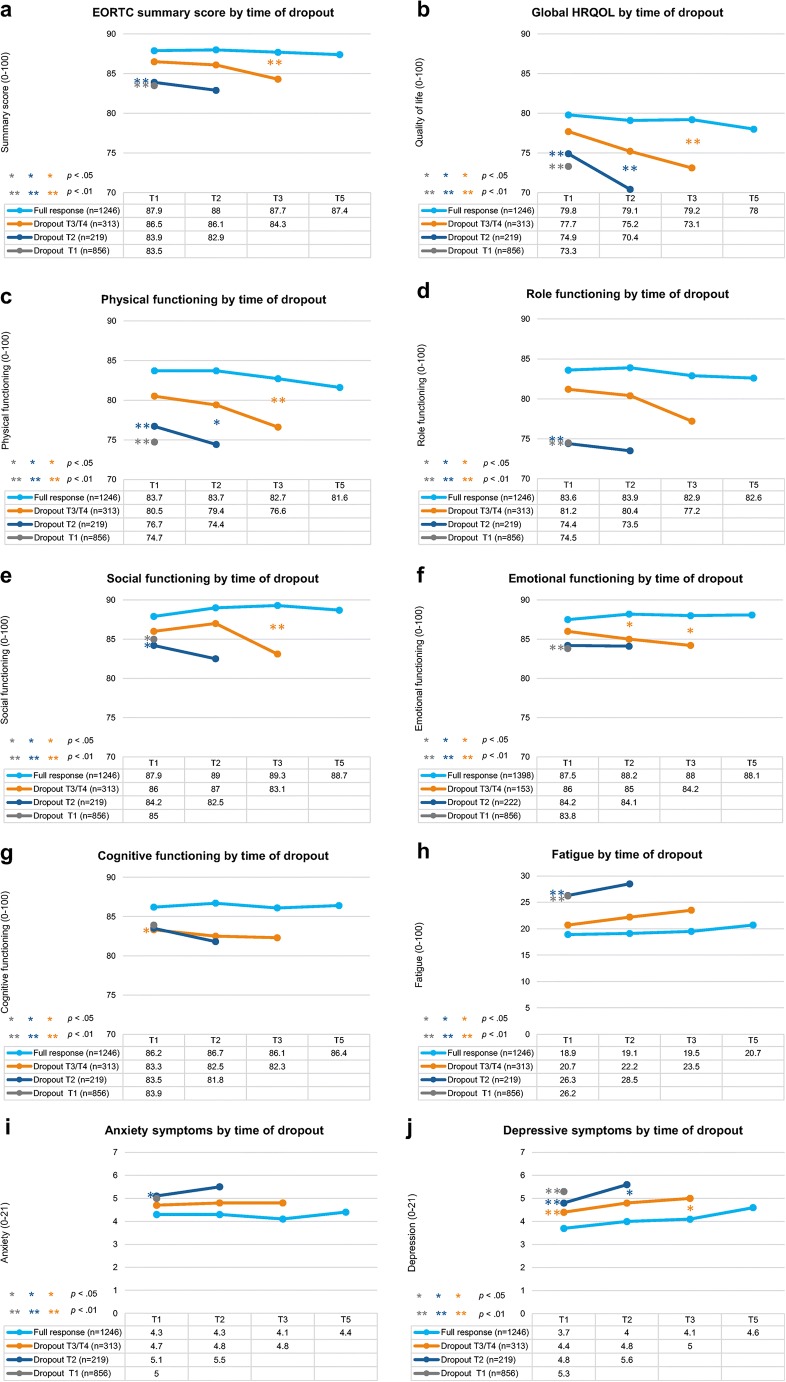
Unadjusted mean functioning scores on the EORTCQLQ-C30 (a– h) and anxiety and depressive symptoms on the HADS (i, j) according to time of dropout (range: 0–100 and 0–21, respectively). Note: EORTC QLQ-C30 scales range from0 to 100; higher scores reflect better perceived HRQOL. HADS scales range from 0 to 21; higher scores reflect higher prevalence of anxiety and depressive symptoms. p values indicate significant group differences between slopes and baseline scores compared with full responders in multilevel mixed models adjusted for time, age, sex, socioeconomic status, education, marital status, comorbidity, disease stage, and treatment received

