# Supplementary material for: Cancer survivors who fully participate in the PROFILES registry have better health-related quality of life than those who drop out
Source: J Cancer Surviv. 2019 Sep 6;13(6):829–39. doi: 10.1007/s11764-019-00793-7 (PMC6881419; doi:10.1007/s11764-019-00793-7)
Supplement: Supplementary file 1 — (DOCX 15 kb) [file 11764_2019_793_MOESM1_ESM.docx]

**Table S1.** Baseline functioning and HRQOL scores by time of dropout

|  | | Dropout T1  *N* = 856 | Dropout T2  *N* = 219 | Dropout T3/T4  *N* = 313 | Full responders  *N* = 1246 | *p*-value |
| --- | --- | --- | --- | --- | --- | --- |
| Summary score, M (SD) | 83.5 (15.2)^a^ | 83.9 (14.3)^a^ | 86.5 (13.4)^b^ | 87.9 (12.3)^b^ | <.001 |  |
| Global HRQOL, M (SD) | 73.3 (21.3)^a^ | 74.9 (17.8)^a,b^ | 77.7 (19.0)^b,c^ | 79.8 (17.3)^c^ | <.001 |  |
| Physical functioning, M (SD) | 74.7 (22.9)^a^ | 76.7 (20.8)^a^ | 80.5 (20.7)^b^ | 83.7 (18.1)^b^ | <.001 |  |
| Role functioning, M (SD) | 74.5 (30.0)^a^ | 74.4 (29.7)^a^ | 81.2 (27.0)^b^ | 83.6 (24.6)^b^ | <.001 |  |
| Social functioning, M (SD) | 85.0 (24.1)^a^ | 84.2 (24.4)^a^ | 86.0 (23.7)^a^ | 87.9 (20.5)^a^ | <.001 |  |
| Emotional functioning, M (SD) | 83.8 (21.0)^a^ | 84.2 (20.3)^a,b^ | 86.0 (19.7)^a,b^ | 87.5 (17.9)^b^ | <.001 |  |
| Cognitive functioning, M (SD) | 83.9 (20.7)^a^ | 83.5 (20.0)^a^ | 83.3 (21.8)^a^ | 86.2 (19.8)^a^ | .02 |  |
| Fatigue, M (SD) | 26.2 (26.1)^a^ | 26.3 (24.9)^a^ | 20.7 (22.3)^b^ | 18.9 (21.3)^b^ | <.001 |  |
| Pain, M (SD) | 18.2 (25.4)^a^ | 18.2 (26.2)^a^ | 16.6 (24.7)^a^ | 15.0 (23.1)^a^ | .02 |  |
| Nausea, M (SD) | 5.0 (13.9)^a^ | 4.3 (12.0)^a,b^ | 4.2 (12.2)^a,b^ | 2.8 (9.9)^b^ | <.001 |  |
| Depression, M (SD) | 5.3 (4.2)^a^ | 4.8 (3.8)^a,b^ | 4.4 (3.6)^b^ | 3.7 (3.3)^c^ | <.001 |  |
| Anxiety, M (SD) | 5.0 (4.0)^a,b^ | 5.1 (3.8)^a^ | 4.7 (3.9)^a,b^ | 4.3 (3.6)^b^ | <.001 |  |

Note: *p*-values report overall ANOVA tests. ^a-c^Means in a row without a common superscript letter differed (*p* <0.05) in post hoc analysis using Tukey’s method. EORTC QLQ-C30 scales range from 0 to 100; higher scores reflect better perceived HRQOL. HADS scales range from 0 to 21; higher scores reflect higher prevalence of anxiety and depressive symptoms.
